# Effect of Influenza Vaccination on the Disease Severity and Viral Load Among Adult Outpatients and Inpatients

**DOI:** 10.3390/vaccines13101046

**Published:** 2025-10-10

**Authors:** Alexander Domnich, Vincenzo Paolozzi, Giada Garzillo, Andrea Orsi, Giancarlo Icardi

**Affiliations:** 1 Hygiene Unit, San Martino Polyclinic Hospital-IRCCS for Oncology and Neurosciences, 16132 Genoa, Italy; andrea.orsi@unige.it (A.O.); icardi@unige.it (G.I.); 2 Department of Health Sciences (DISSAL), University of Genoa, 16132 Genoa, Italy; vincenzo.paolozzi@edu.unige.it (V.P.); 4789907@studenti.unige.it (G.G.); 3 Interuniversity Research Centre on Influenza and Other Transmissible Infections (CIRI-IT), 16132 Genoa, Italy

**Keywords:** influenza, vaccination, influenza vaccines, vaccine effectiveness, severity, breakthrough infections, adults, older adults

## Abstract

**Background**: Some studies suggest that, thanks to the mechanisms of immune-mediated attenuation, influenza vaccination reduces severity of influenza illness in breakthrough infections. This study aimed to assess whether influenza vaccination attenuates severity of laboratory-confirmed influenza among Italian adults. **Methods**: This secondary analysis included all influenza cases detected during respiratory surveillance studies conducted in outpatient and inpatient settings in Genoa (Italy), throughout the 2023/2024 and 2024/2025 seasons. Here, we compared viral load and the count of influenza-related symptoms in outpatients, alongside all-cause in-hospital mortality and radiologically confirmed pneumonia in inpatients, between vaccinated and unvaccinated adults. **Results**: The study included 188 influenza cases diagnosed in primary care and 281 influenza cases identified among inpatients. Of these, 37.2% and 31.7%, respectively, were vaccinated, constituting breakthrough infections. Compared to unvaccinated adults, vaccinated outpatients had a slightly lower viral load (difference in cycle threshold values of 1.36 corresponding to about 0.51 log_10_ reduction in the number of copies/mL; *p* = 0.077), primarily driven by influenza A(H1N1)pdm09. Vaccinated outpatients also reported 9% fewer influenza-related symptoms than unvaccinated counterparts [prevalence ratio 0.91; 95% confidence interval (CI): 0.84, 0.99]. Among hospitalized older adults, influenza vaccination was associated with 64% reduced odds of in-hospital death (odds ratio 0.36; 95% CI: 0.12, 0.94). Conversely, no association between vaccination and development of pneumonia was found. **Conclusions**: This study corroborates the idea that influenza vaccination attenuates disease severity in breakthrough infections. These effects are, however, dependent on the measure of severity used.

## 1. Introduction

Each year, seasonal influenza affects nearly 10% of adults, about half of whom are symptomatic [1]. Although most infections are mild, up to 650,000 influenza-related respiratory deaths are estimated to occur annually [2]. Older adults, young children, individuals with co-morbidities and pregnant women are at greater risk of developing severe disease resulting in hospitalization or death [2,3]. Even mild uncomplicated influenza cases in younger adults lead to a considerable socioeconomic burden, primarily driven by absenteeism costs [4].

Annual influenza vaccination (IV) is a globally recognized cornerstone of public health, whose benefits span a broad spectrum of health outcomes including respiratory illness, hospitalization for influenza and pneumonia, and all-cause mortality [2,5]. Although the available inactivated influenza vaccines (IIVs) reduce the risk of severe outcomes, which is the primary goal of IV programs, their effectiveness against symptomatic infection is often referred to as suboptimal, ranging from 10% to 60% [6]. In other words, breakthrough infections, which refer to laboratory-confirmed cases in vaccinated people, are not rare. This high variability of influenza vaccine effectiveness (IVE) is determined by a plethora of factors related to the virus, vaccinee, and IIV itself [7].

Neither influenza infection nor IV confers sterilizing immunity that blocks infection by preventing viral replication within the host [8]. The major goal of effective vaccines is therefore to attenuate disease severity, especially in the most vulnerable populations. The concept of immune-mediated attenuation implies that pre-existing immunity or anamnestic recall in response to virus re-exposure diminishes disease severity, even if the immune response is insufficient to prevent breakthrough infections [8]. Importantly, it has been suggested [9] that the full impact of vaccination is typically greater than the impact assessed by a point estimate of IVE. In this regard, the question of whether IV may attenuate disease severity in breakthrough infections is integral to understanding the full benefit.

A recent systematic review and meta-analysis [10] identified a total of 38 primary studies that aimed to assess whether IV attenuates the severity of breakthrough influenza infections. Out of 21 severity indicators identified, five could be meta-analyzed. These pooled analyses, in which the association was measured by summary odds ratios (ORs), showed mixed results. In particular, IV was associated with 26% reduction in the odds of intensive care unit (ICU) admission [OR 0.74; 95% confidence interval (CI): 0.58, 0.93], 31% reduction in the odds of death (OR 0.69; 95% CI: 0.52, 0.92), and 45% reduction in the odds of fever among children (OR 0.55; 95% CI: 0.42, 0.71). Conversely, the association was not statistically significant for the indicators of clinical diagnosis of pneumonia among inpatients (OR 0.92; 95% CI: 0.82, 1.04) and hospital encounter following outpatient influenza episode (OR 0.60; 95% CI: 0.28, 1.28). This review emphasized a sparsity of the available studies (especially those on the association between IV and subjective severity indicators as well as between IV and functional impact of mild illness), and lack of conceptual clarity, standardization and imprecise measurement of what constitutes severe influenza disease [10]. Similarly, Patel and colleagues [8] called for a standardization of severity scores to account for heterogeneity in clinical presentation of influenza illness. Intrigued by the heterogeneity of effect estimates and driven by the lack of Italian data [10], this study sought to compare features and severity of medically attended laboratory-confirmed influenza episodes among Italian adult outpatients and inpatients during the 2023/2024 and 2024/2025 seasons, according to their IV status.

## 2. Materials and Methods

### 2.1. Study Overview

This study represents a secondary analysis of a two-season study conducted in primary care [11,12] and two hospital-based studies conducted during two recent influenza seasons [13,14]. Both outpatient and inpatient studies were conducted during the 2023/2024 and 2024/2025 influenza seasons in Genoa (Italy). All studies were approved by the Ethics Committee of Liguria Region (#166/2023, id 13094; #397/2023, id 13354).

The outpatient study had a cluster-randomized design and aimed to compare detection rates of respiratory syncytial virus (RSV) among adults meeting European criteria [15] for acute respiratory infection (ARI) or influenza-like illness (ILI) and to assess the natural history and economic consequences of RSV. To this end, up to 36 general practitioners (GPs) were randomized in two groups: the first group enrolled subjects meeting case definition of ARI [sudden onset of at least one respiratory symptom (cough, sore throat, shortness of breath, coryza) and GP’s judgment of an underlying infection], while the second group enrolled those meeting the European ILI [sudden onset of at least one systemic (fever or feverishness, malaise, headache, myalgia) and at least one respiratory (cough, sore throat, shortness of breath) symptom] criteria. Adults aged ≥50 years who met the case definitions of ARI or ILI were potentially eligible. Individuals were excluded if they developed symptoms > 7 days before the visit, were residents of nursing homes or were positive for RSV in the current season. All individuals underwent a nasopharyngeal swab and were systematically tested for influenza virus (sub)types and other common respiratory pathogens. Full details of this outpatient study can be accessed elsewhere [11,12].

The inpatient studies were conducted in a large tertiary care research hospital with the aim of quantifying IVE against hospitalization for laboratory-confirmed influenza. Both seasonal studies were designed as retrospective test-negative case–control studies, in which patients who molecularly tested positive for influenza virus were cases, while those testing negative were deemed controls. Adult individuals aged ≥18 years who had accessed the hospital emergency department (ED) with a subsequent hospitalization and a prescription for at least one real-time polymerase chain reaction (RT-PCR) test for the detection of influenza within five days following the ED visit were potentially eligible. Subjects were excluded if they had a laboratory-confirmed current season influenza before the ED visit or if their IV status could not be verified. Further aspects of the hospital-based studies can be accessed elsewhere [13,14].

In line with the objective of the present analysis, we extracted data on subjects who tested positive for any influenza virus. Subjects were excluded if they received IIV 1–13 days before the onset of symptoms or ED access [16].

### 2.2. Study Outcomes

Two influenza-related endpoints were considered for the outpatient study, namely viral load and total symptom score. Viral load was proxied using cycle threshold (Ct) values, which represent the number of amplification cycles required for the fluorescent signal to cross a threshold of background noise. Ct values are inversely related to viral load: lower Ct values indicate a higher viral load [17]. Samples with Ct values ≥ 30 were considered to have a low viral load [18,19].

The total symptom score was defined as the sum of individual symptom/sign scores (0 = absent; 1 = present) collected by GPs during the initial visit. A similar approach was used in previous research [20,21]. In particular, GPs collected a total of nine systemic (fever/feverishness, shivering, headache, myalgia, arthralgia, malaise, decreased appetite, nausea, diarrhea) and 11 respiratory (cough, sputum production, dyspnea, tachypnea, rhonchi, wheezing, desaturation, sore throat, coryza, altered smell, altered taste) signs or symptoms for each outpatient. The total symptom score could range from 1 to 20, since the presence of at least one respiratory symptom was among the inclusion criteria (i.e., qualifying respiratory symptoms for the ARI/ILI criteria defined earlier in the text).

Radiologically confirmed pneumonia and in-hospital mortality constituted the endpoints of the inpatient study. The former was defined as pneumonia diagnosed through chest X-ray or other imaging, as documented in patient files. In-hospital mortality was defined as death from any cause registered during the current hospitalization episode, regardless of the length of stay.

### 2.3. Laboratory Diagnosis of Influenza

Nasopharyngeal swab specimens collected in both outpatient and inpatient studies were eluted in a transport medium and tested in fresh using the same RT-PCR protocol. Specifically, samples were tested using the Allplex Respiratory Panels 1–4 and Allplex SARS-CoV-2/FluA/FluB/RSV (Seegene Inc., Seoul, Republic of Korea). The former multiplex kit detects a broad range of respiratory pathogens, including the following targets: influenza A, A/H1, A/H1pdm09, A/H3 and B; RSV A and RSV B; adenovirus; enterovirus; metapneumovirus, parainfluenza viruses 1, 2, 3 and 4; bocaviruses 1–4; seasonal coronaviruses 229E, NL63 and OC43; human rhinovirus; *Streptococcus pneumoniae*; *Bordetella pertussis*; *Bordetella parapertussis*; *Chlamydophila pneumoniae*; *Hemophilus influenzae*; *Legionella pneumophila* and *Mycoplasma pneumoniae*. Additionally, the Allplex SARS-CoV-2/FluA/FluB/RSV was used to detect SARS-CoV-2 gene targets. Testing was performed according to the manufacturer’s instructions. Briefly, nucleic acids underwent extraction and purification using the STARMag 96 × 4 Universal Cartridge Kit (Seegene Inc., Seoul, Republic of Korea). This process was automated on the STARlet liquid handling workstation (Seegene Inc., Seoul, Republic of Korea), involving the extraction of 200 µL of sample and subsequent elution with 100 µL of the provided elution buffer. The RT-PCR reactions were run on a CFX96 system (Bio-Rad Laboratories; Hercules, CA, USA) utilizing the Allplex kits, as detailed in the manufacturer’s specifications. Each reaction incorporated 8 µL of the extracted nucleic acids within a 25 µL final volume.

Using eight serial dilutions (from 250 to 50,000 copies/mL) of a reference material (AccuPlex SARS-CoV-2, Flu A/B and RSV Verification Panel; SeraCare, Milford, MA, USA), we estimated (via log-linear regression) that each 1-unit increase in Allplex Ct values for influenza A is associated, on average, with a 0.37 log_10_ viral load reduction in the number of copies/mL.

### 2.4. Influenza Vaccination Status

Current season IIV was the independent variable of interest. Subjects were considered vaccinated if IIV administration took place at least 14 days before symptom onset or ED visit [16]. With regard to adult Italian population (≥18 years), IV was recommended and fully reimbursed for older adults aged ≥60 years, subjects of any age with underlying health conditions, pregnant women and some other categories [22].

Vaccination status was ascertained in a local electronic immunization registry, where recording of IIV receipt is mandatory for reimbursement purposes, and, therefore, the risk of exposure misclassification is low. This registry has been validated and extensively used in IVE and surveillance studies [13,14]. During the study period, most IIVs administered in the study area were standard-dose egg-based, adjuvanted, and high-dose IIVs. Exposure to a cell-based IIV was limited. Moreover, the adjuvanted IIV was preferentially recommended to older adults aged 60/65–79 years (65–79 years in the 2023/2024 season and 60–79 years in the 2024/2025 season), while the high-dose IIV was preferentially recommended to subjects aged ≥80 years. However, these recommendations were not mandatory, and any age-appropriate vaccine could be administered to older adults [13,14].

### 2.5. Study Variables and Potential Confounders

The following independent variables were considered as potential confounders: sex; age; presence of co-morbidities (cardiovascular, respiratory, renal, and hepatic diseases, diabetes, immunosuppression including cancer), current smoking, and swab delay (number of days between swab collection and symptom onset/ED visit).

### 2.6. Data Analysis

For descriptive statistics, binary and categorical variables were reported as proportions with Clopper-Pearson’s exact 95% CIs. Continuous variables were reported as means with standard deviations (SDs) and/or medians with interquartile ranges (IQRs). Violin plots were used to visualize Ct value distributions by IIV status.

The association between IV status and the continuous variable of Ct was modeled via linear regression. Lilliefors (Kolmogorov–Smirnov) test was used to check normality of the Ct value distribution. The base-case linear models considered any influenza virus (sub)type. A subgroup analysis by influenza virus subtypes was also conducted.

Quasi-Poisson models were applied to study the association between IV and the count of symptoms constituting the total symptom score. The effect size was expressed as prevalence ratios (PRs). Considering that the distribution of the total symptom score approached a normal distribution, we performed a sensitivity analysis by treating the dependent variable as a continuous variable [20].

To account for a relatively small number of events among inpatients, the association between IV and binary outcomes of in-hospital mortality and radiologically confirmed pneumonia was modeled by applying Firth’s bias-reduced logistic regression [23,24]. Since the population of inpatients was skewed towards older adults aged ≥65 years (and most events occurred in older adults), this analysis was performed in the entire adult population aged ≥18 years and in that of older adults. Owing to the paucity of cases and events, no separate analysis for working-aged (18–64 years) adults could be conducted.

For all aforementioned regression specifications, univariable models were first evaluated. Potential predictors with *p* < 0.2 at univariable analysis were included in the multivariable model [25] to account for potential confounders.

Statistical analysis was performed in R software, version 4.5.0 (R Foundation for Statistical Computing, Vienna, Austria).

## 3. Results

### 3.1. Outpatient Studies

#### 3.1.1. Characteristics of the Study Population

Out of 190 adult outpatients aged ≥50 years who tested positive for influenza in the 2023/2024 and 2024/2025 seasons, 188 were included in the analysis. Two subjects were excluded because they were vaccinated 1–13 days before the onset of symptoms. As shown in Table 1, the mean age of patients was 64.5 (SD 11.0) years, and women slightly prevailed (54.3%). About one half (48.4%) of participants had at least one co-morbidity, of which cardiovascular and respiratory conditions were the most prevalent. Most detections belonged to influenza type A, with A(H1N1)pdm09 (51.6%) more prevalent than A(H3N2) (39.4%). Influenza B accounted for only 8.5%. Additionally, there was one (0.5%) A(H1N1)pdm09 and B co-detection. A total of 26.1% of patients had ≥1 non-influenza viral or bacterial co-detections, with *Streptococcus pneumoniae* and/or *Hemophilus influenza* as the most common. Full RT-PCR results are reported in the Supplementary Materials, Table S1. The prevalence of breakthrough infections was 37.2% (95% CI: 30.3%, 44.6%). Most vaccinated subjects (*N* = 70) were vaccinated with either standard-dose egg-based (45.7%) or adjuvanted (41.4%) IIVs, while the number of recipients of the high-dose (11.4%) and cell-based (1.4%) IIVs was limited. Cases were caused by A(H1N1)pdm09 (51.4%; 36/70), A(H3N2) (42.9%; 30/70) and B (5.7%; 4/70) viruses.

#### 3.1.2. Association Between Influenza Vaccination and Influenza Viral Load

**Figure 1:**
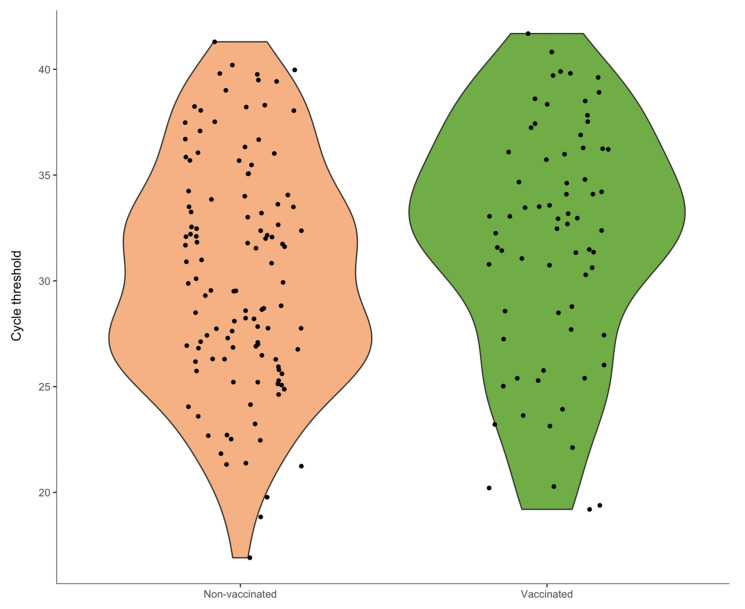
Distribution of cycle threshold values according to influenza vaccination status (*N* = 188). Note: These violin plots show distributions of cycle threshold (Ct) values between non-vaccinated (left graphic) and vaccinated (right graphic) adult outpatients. Lower Ct values correspond to higher viral loads. A skew toward higher Ct values was observed in the vaccinated cohort compared with the non-vaccinated cohort, notwithstanding a considerable common range of values.

The distribution of Ct values was approximately normal (*p* = 0.15) with a mean of 30.9 and a median of 31.5. Vaccinated individuals had higher average Ct values than non-vaccinated adults (31.8 vs. 30.3), corresponding to an approximate 0.58 log_10_ reduction in the number of copies/mL. Despite a considerable overlap between vaccinees and non-vaccinees, the distribution of Ct values for the former group was shifted towards higher values (Figure 1). Indeed, the proportion of samples with low viral load (Ct ≥ 30) was 70.0% (49/70) among vaccinated adults and 49.2% (58/118) among unvaccinated adults.

In linear regression analysis, the number of days between the onset of symptoms and swab was the strongest predictor: each additional day was associated with an approximately 1.5-unit increase in Ct values. In a multivariable model, vaccination was associated with a 1.36-unit (95% CI: −0.14, 2.85) increase in Ct values, which corresponds to about 0.51 log_10_ reduction in the number of copies/mL. Compared with the A(H1N1)pdm09 subtype, Ct values for A(H3N2) were on average 1.69 units lower (Table 2).

Subgroup analysis by influenza A subtypes showed a greater viral load reduction for A(H1N1)pdm09 than for A(H3N2). After adjustment for swab delay, the estimated Ct values for A(H1N1)pdm09 among vaccinated individuals were 2.10 (95% CI: 0.13, 4.06) units lower (*p* = 0.039). For A(H3N2), the difference (0.98; 95% CI: −1.45, 3.41) was not significant (*p* = 0.43). Due to the small number of influenza B cases, a separate analysis was not feasible.

#### 3.1.3. Association Between Influenza Vaccination and Symptomatic Profile

**Figure 2:**
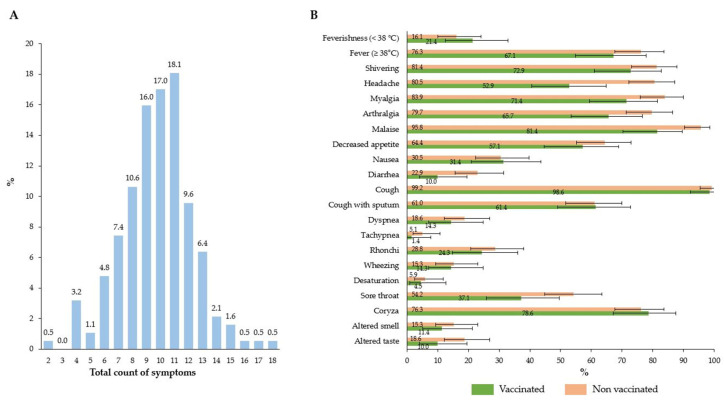
Distribution of the count of symptoms among outpatients (**A**) and prevalence of single symptoms among vaccinated and non-vaccinated outpatients (**B**) (*N* = 188). Note: The vertical bar chart on the left panel (**A**) shows that most outpatient adults reported 8–11 symptoms (maximum 20) associated with their episode of acute respiratory infection. The horizontal bar chart on the right panel (**B**) compares frequencies of single signs and symptoms between vaccinated and non-vaccinated individuals. Although most 95% confidence intervals for individual symptoms overlap, vaccinated individuals tended to report fewer symptoms.

Among 188 outpatients (Figure 2), the median and mean total symptom scores were 10 [IQR: 8, 11] and 9.8 (SD 2.5), respectively (Figure 2A). Vaccinated subjects tended to report fewer symptoms than unvaccinated counterparts, but 95% CIs generally overlapped. However, as shown by non-overlapping 95% CIs, the prevalence of headache (52.9% vs. 80.5%) and malaise (81.4% vs. 95.8%) was lower among vaccinees (Figure 2B).

In the univariable analysis, vaccinees were expected to report 14% (PR 0.86; 95% CI: 0.80, 0.93) fewer symptoms than their non-vaccinated counterparts. After adjustment, the effect size decreased to 9% but was still different from the null (*p* = 0.026) (Table 3). When the total symptom score was treated as a continuous variable and a multivariable linear model was reapplied, IV was associated with 0.92 (95% CI: 0.12, 1.71) fewer symptoms (Supplementary Material, Table S2).

### 3.2. Inpatient Studies

#### 3.2.1. Characteristics of the Study Population

Of 285 influenza cases among hospitalized adults available, four (1.4%) were excluded because vaccinated 1–13 days before the ED access. Principal characteristics of 281 inpatients are reported in Table 4. Briefly, most (70.5%) subjects were ≥65 years (mean age 72.5 years), and males slightly prevailed (52.0%). A total of 79.3% had ≥1 underlying condition, of which cardiovascular disease (64.4%), respiratory conditions (23.5%), and diabetes (17.1%) were the most frequent. One third (31.7%) of patients had been previously vaccinated. Among vaccinees (*N* = 89), 40.4%, 34.8%, 23.6% and 1.1% were immunized with high-dose, standard-dose egg-based, adjuvanted and cell-based IIVs, respectively. The distribution of virus (sub)types was as follows: 59.1%, 36.7% and 4.3% of cases were caused by A(H1N1)pdm09, A(H3N2) and B, respectively.

#### 3.2.2. Association Between Influenza Vaccination and Severe Influenza-Related Outcomes

Among 281 inpatient adults, 24 died during their hospital stay (case-fatality rate 8.5%; 95% CI: 5.5%, 12.4%). Of 24 deaths, all but one (95.8%) were ≥65 years, and only five (20.8%) were vaccinated. Indeed, among older adults, the in-hospital mortality rate was 2.8% (5/80) among vaccinees and 15.3% (18/118) among non-vaccinees (*p* = 0.040). When adjusted, IV was associated with a 64% reduction (OR 0.36; 95% CI: 0.12, 0.94) in the odds of in-hospital mortality (Table 5).

Ninety-eight (36.2%; 95% CI: 29.3%, 40.8%) subjects had a radiologically confirmed pneumonia. Prevalence of pneumonia was lower in vaccinees (28.1%; 25/89) than non-vaccinees (38.0%; 73/192), but the difference was not statistically significant in both univariable (*p* > 0.1) and multivariable (*p* > 0.4) analyses (Table 5). Full regression results are reported in the Supplementary Materials, Tables S3 and S4.

## 4. Discussion

While the monitoring of seasonal IVE is essential, the full value of IV extends beyond what IVE alone describes [9,26,27]. In this combined analysis of outpatient and inpatient data collected during two recent influenza seasons, we showed that IV reduces both the number of symptoms among adults with breakthrough infections managed in primary care and mortality among hospitalized adults with laboratory-confirmed influenza. On the other hand, we were unable to demonstrate significant effects of IV on the development of pneumonia and viral load. Our study, therefore, provides additional data on the association between IV and severity of breakthrough infections, which were claimed to be sparse [10], rudimentary and conflicting [8].

In this study, there was little evidence of a lower viral load in symptomatic outpatients who received IV. Effects of vaccination on the viral load of respiratory viruses are largely unknown and inconclusive. Most observational studies focused on SARS-CoV-2 and COVID-19 vaccination. An English study [28] reported that adults vaccinated with either one dose or two doses of the COVID-19 vaccine showed on average higher Ct values (Ct difference of 1.21–1.63; *p* < 0.05) than unvaccinated individuals. A large Israeli study [29] on 16,553 SARS-CoV-2 infections caused by the Delta variant estimated that compared with unvaccinated individuals, those fully vaccinated with BNT162b2 had higher Ct values (Ct difference 4.6; 95% CI: 2.2, 6.9) 7–30 days post-vaccination. This difference waned over time to 0.6 (95% CI: 0.05, 1.12) about two months post-vaccination and almost disappeared six months post-vaccination. However, this decay was overturned when a booster dose was administered, and the difference turned significant up to 34 days post-booster dose [29]. On the other hand, one cohort study [30] found similar (*p* = 0.93) median SARS-CoV-2 Ct values between vaccinated (19.2) and unvaccinated (18.8) individuals at diagnosis, but viral loads decreased faster among vaccinees. Still another study conducted in the US reported [31] opposite results: median Ct values among vaccinees (20.5–22.2) were significantly lower than those among non-vaccinees (22.1–23.5). With regard to influenza, the available evidence, especially for older adults, is more limited. One Spanish study [19] revealed that the viral load was higher among unvaccinated adults when compared with their vaccinated counterparts (Ct 25.17 vs. 27.58, *p*  =  0.004). Conversely, Spencer et al. [18] reported that the proportion of samples with low Ct values was comparable between vaccinated and non-vaccinated subjects aged ≥3 years. Available pediatric studies [32,33] highlighted no association between viral load and IV status. These inconsistent results appear to be driven by a number of study-specific factors, such as age and virus (sub)type distributions, setting, sample size, and methods used to collect data on the date of symptom onset.

We then established that vaccinated outpatients aged ≥50 years had, on average, 9% fewer symptoms than their unvaccinated counterparts. Although a direct comparison is hindered by differences in the construction of summary symptom severity scores, our results are consistent with findings from previous studies conducted in the US [20,21]. Specifically, one cross-sectional survey [20] analyzed the association between IV status and ARI symptom severity score, which was defined as the sum of self-rated scores (from 0 absent to 3 severe) for eight symptoms (nasal congestion, sore throat, cough, wheezing, feverishness, fatigue, headache, muscle aches). Out of a maximum score of 24 indicating the highest severity, the average scores were 12.3 (SD 4.0) for adults aged 50–64 years and 10.8 (SD 4.2) for those aged ≥65 years. When stratified by IV status, the mean severity score among vaccinated adults aged ≥65 years was about 5 points lower (*p* < 0.001) than among unvaccinated older adults. This reduction was much lower (approximately 1 point) among younger adults aged 50–64 years. Another US study [21] used a similar score construction method, in which 21 different symptoms were rated on a 4-point ordinal scale (from 0 none to 3 severe), but data were collected longitudinally for the first seven days. In their multivariable analysis performed on the dataset restricted to A(H3N2) infections, vaccinees showed significantly lower total symptom scores than non-vaccinees on most days after symptom onset. For example, on the first day, the median score was 25 among non-vaccinees, which was about twice (12) that of vaccinees. However, there were no statistically significant differences between vaccinees and non-vaccinees for A(H1N1), B, or any influenza virus [21].

In the hospital setting, we observed 42–64% (depending on the adjustment strategy and age of inpatient) reduction in the odds of all-cause mortality among vaccinees compared to non-vaccinees. The analogous reduction (by 9–36%) in terms of the odds of radiologically confirmed pneumonia was not statistically significant. These findings align with the pooled analysis by Ferdinands et al. [10] who reported a significant association between IV and death but not between IV and clinically diagnosed pneumonia (pooled OR 0.92; 95% CI: 0.82, 1.04). In our study, the magnitude of reduction in death was generally greater than the pooled OR (0.69; 95% CI: 0.52, 0.92) reported in the aforementioned meta-analysis. With a moderate level of heterogeneity in that meta-analysis (*I*^2^ = 42%) [10], single-study ORs varied from 0.21 (95% CI: 0.05, 0.97) [34] to 1.25 (95% CI: 0.54, 2.90) [35]. Indeed, regarding older adults aged ≥65 years, our effect size (aOR 0.36; 95% CI: 0.12, 0.94) approaches that reported in a US study conducted during the 2013/2014 influenza season (aOR 0.39; 95% CI: 0.17, 0.66). That study also did not find a meaningful association between IV and radiologically confirmed influenza in all age groups. Notably, similar to our study, the 2013/2014 US season was characterized by a predominance of the A(H1N1)pdm09 subtype [34]. Altogether, these observations suggest that the association between IV and mortality may vary by season and/or predominant virus (sub)type. Another reason for a comparatively higher effect size in our study can be ascribed to a widespread use of the high-dose and adjuvanted IIVs, which were preferentially recommended to older adults aged 60/65–79 and ≥80 years, respectively. In a three-season US cohort study [36], compared with unvaccinated older adults aged ≥65 years, those immunized with the high-dose IIV showed a significant mortality reduction during the 2016/2017 (29%; 95% CI: 9%, 45%), 2017/2018 (17%; 95% CI: 5%, 28%) and 2018/2019 (27%; 95% CI: 10%, 41%). The analogous percent reductions in the cohort of subjects immunized with the standard-dose IIV were significant only in the 2016/2017 (25%; 95% CI: 2%, 43%), but not in the 2017/2018 (−3%; 95% CI: −23%, 14%) and 2018/2019 (21%; 95% CI: −9%, 43%) seasons.

In a nutshell, the available evidence [10], including the present study, supports the idea that even in cases when IV fails to prevent infection, it is still able to attenuate severity of influenza illness. Furthermore, IV appears to be more effective for the most severe outcomes, while measures used to approximate disease severity are seemingly a major driver of some conflicting results and variations in the reported effect sizes. In this regard, one Delphi study [37] demonstrated that IVE studies should also include severe influenza-related outcomes, such as deaths, because these latter endpoints would exercise a greater impact on decision makers.

This study suffers from several important shortcomings. First, despite numerically higher Ct values observed in vaccinated versus unvaccinated adults, their practical significance (e.g., in terms of reduced infectiousness or shorter duration of viral shedding) remains unclear, even without considering the observed type I error (*p* = 0.077). The cross-sectional study design relied on a single swab specimen collected during the initial GP visit and did not allow us to establish whether vaccinees have a different pattern of viral load kinetics than non-vaccinees. In this regard, longitudinal studies will be of value. Moreover, Ct values are kit-specific and may even vary among different lots of the same RT-PCR kit [38]. Particular attention should be paid when comparing our results with those obtained using alternative RT-PCR assays. Second, although the set of signs and symptoms reported by outpatients was comprehensive, our summary symptom severity score was not formally validated. Severity of single symptoms could only be dichotomized into “present” versus “absent”, thus precluding differentiation between mild, moderate and severe intensity. This lack of granularity may have hidden some patterns of the association between IV and symptom severity. In light of this, the use of validated scales, such as Flu-PRO (patient-reported outcome) [39] or influenza intensity and impact questionnaire FluiiQ [40] would allow for a nuanced differentiation between mild, moderate, and severe cases and eventually for a more granular association between symptom severity and IV status. Third, a comparatively low number of cases and, especially, some outcomes like influenza-related deaths led to imprecise point estimates with wide 95% CIs. This high degree of uncertainty regarding the true effect size warrants cautious interpretation and limits the definitive conclusions. For the same reason, some subgroup analyses (e.g., for working-aged adults and influenza B) could not be performed. Future large-scale, age-stratified analyses are needed to provide a more granular view of the association between IV and various indicators of disease severity. Fourth, although some research advocates a parsimonious approach to confounder adjustment in test-negative case–control studies [41], our results may be subject to residual confounding. Finally, as no clinical case definition was required for the hospital-based studies, these latter may be subject to selection bias (e.g., RT-PCR tests prescribed to asymptomatic individuals for screening purposes, systematic differences in RT-PCR prescription between vaccinated and non-vaccinated subjects).

## 5. Conclusions

In conclusion, our findings support the concept of immunoattenuation [8] induced by IV, which postulates that the severity of influenza illness among individuals with breakthrough infections is reduced. This is, indeed, biologically plausible, as immunological memory may allow the adaptive immune response to reduce virus replication and speed up the clearance of infected cells [10]. In the context of high variability of IVE by season, virus (sub)type, target groups and other factors, policy makers should be informed about these additional benefits of annual IV.

## Figures and Tables

**Table 1:** Characteristics of adult outpatients who tested positive for influenza (*N* = 188).

Variable	Level	*n* (%)
Sex	Female	102 (54.3)
	Male	86 (45.7)
Age, years	Mean (SD)	64.5 (11.0)
Swab delay, days	Mean (SD)	3.3 (1.5)
Season	2023/2024	57 (30.3)
	2024/2025	131 (69.7)
Presence of co-morbidities	≥1	91 (48.4)
	Cardiovascular	64 (34.0)
	Respiratory	25 (13.3)
	Diabetes	10 (5.3)
	Hepatic	6 (3.2)
	Renal	3 (1.6)
	Cancer/immunosuppression	21 (11.2)
Smoking	Yes	24 (12.8)
Influenza virus (sub)type	A(H1N1)pdm09	97 (51.6)
	A(H3N2)	74 (39.4)
	B	16 (8.5)
	A(H1N1)pdm09 + B	1 (0.5)
Non-influenza virus co-detection	Yes	19 (10.1)
Bacterial co-detection	Yes	34 (18.1)
Influenza vaccination	Yes	70 (37.2)

SD: standard deviation.

**Table 2:** Association between influenza cycle thresholds (lower cycle threshold corresponds to higher viral load) and potential predictors (*N* = 188).

Variable	Univariable Models		Multivariable Model	
	*b* (95% CI)	*p*	*b* (95% CI)	*p*
Influenza vaccination	1.56 (−0.06, 3.19)	0.061	1.36 (−0.14, 2.85)	0.077
Sex (male vs. female)	−0.22 (−1.81, 1.37)	0.79	–	–
Age (10-year increase)	0.19 (−0.53, 0.91)	0.60	–	–
Swab delay (1-day increase)	1.46 (0.97, 1.94)	<0.001	1.49 (1.01, 1.97)	<0.001
Season (2024/2025 vs. 2023/2024)	0.74 (−0.98, 2.46)	0.40	–	–
≥1 co-morbidity	0.40 (−1.19, 1.98)	0.62	–	–
Cardiovascular disease	1.07 (−0.59, 2.74)	0.21	–	–
Respiratory disease	1.04 (−1.29, 3.37)	0.38	–	–
Cancer/immunosuppression	−0.26 (−2.78, 2.25)	0.84	–	–
Smoking	−0.16 (−2.54, 2.21)	0.89	–	–
A(H3N2) vs. A(H1N1)pdm09	−1.14 (−2.82, 0.52)	0.18	−1.69 (−3.21, −0.16)	0.032
B vs. A(H1N1)pdm09 ^1^	−0.41 (−3.26, 2.44)	0.78	−0.75 (−3.36, 1.85)	0.57
Non-influenza virus co-detection, %	−0.84 (−3.47, 1.78)	0.53	–	–
Bacterial co-detection, %	1.16 (−0.89, 3.22)	0.27	–	–

^1^ For one subject tested positive for both A(H1N1)pdm09 and B viruses, the cycle threshold for influenza B (26.85) was considered, with the latter significantly lower than the cycle threshold for A(H1N1)pdm09 (35.61). CI: confidence interval.

**Table 3:** Association between count of signs and symptoms (total symptom score) and potential predictors (*N* = 188).

Variable	Univariable Models		Multivariable Model	
	PR (95% CI)	*p*	PR (95% CI)	*p*
Influenza vaccination	0.86 (0.80, 0.93)	<0.001	0.91 (0.84, 0.99)	0.026
Sex (male vs. female)	0.97 (0.90, 1.04)	0.36	–	–
Age (10-year increase)	0.94 (0.90, 0.97)	<0.001	0.96 (0.92, 0.997)	0.035
Swab delay (1-day increase)	1.01 (0.99, 1.04)	0.38	–	–
Season (2024/2025 vs. 2023/2024)	0.91 (0.84, 0.99)	0.023	0.94 (0.87, 1.01)	0.11
≥1 co-morbidity	0.96 (0.89, 1.04)	0.31	–	–
Cardiovascular disease	0.93 (0.86, 1.00)	0.060	1.00 (0.92, 1.09)	0.93
Respiratory disease	1.06 (0.95, 1.18)	0.32	–	–
Cancer/immunosuppression	1.00 (0.89, 1.12)	0.99	–	–
Smoking	1.10 (0.99, 1.23)	0.076	1.09 (0.98, 1.20)	0.12
A(H3N2) vs. A(H1N1)pdm09	1.00 (0.93, 1.09)	0.91	–	–
B vs. A(H1N1)pdm09	0.94 (0.82, 1.07)	0.35	–	–
Non-influenza virus co-detection, %	0.99 (0.88, 1.12)	0.88	–	–
Bacterial co-detection, %	1.05 (0.95, 1.15)	0.33	–	–
Cycle threshold (10-unit increase)	1.04 (0.97, 1.11)	0.32	–	–

CI: confidence interval; PR: prevalence ratio.

**Table 4:** Characteristics of adult inpatients who tested positive for influenza (*N* = 281).

Variable	Level	*n* (%)
Sex	Female	135 (48.0)
	Male	146 (52.0)
Age, years	Mean (SD)	72.5 (15.7)
	18–64	83 (29.5)
	≥65	198 (70.5)
Swab delay, days	Mean (SD)	2.1 (1.8)
Season	2023/2024	110 (39.1)
	2024/2025	171 (60.9)
Presence of co-morbidities	≥1	223 (79.3)
	Cardiovascular	181 (64.4)
	Respiratory	66 (23.5)
	Diabetes	48 (17.1)
	Hepatic	5 (1.8)
	Renal	30 (10.7)
	Cancer/immunosuppression	27 (9.6)
Influenza virus (sub)type	A(H1N1)pdm09	166 (59.1)
	A(H3N2)	103 (36.7)
	B	12 (4.3)
Influenza vaccination	Yes	89 (31.7)

SD: standard deviation.

**Table 5:** Association between influenza vaccination and severe influenza-related outcomes of in-hospital mortality and radiologically confirmed pneumonia among hospitalized adults, by outcome and age group.

Outcome	Age, Years	OR (95% CI)	*p*	aOR (95% CI)	*p*
In-hospital mortality	≥18	0.58 (0.20, 1.45)	0.26	0.37 (0.12, 0.97) ^1^	0.042
	≥65	0.40 (0.13, 1.01)	0.053	0.36 (0.12, 0.94) ^2^	0.036
Radiologically confirmed pneumonia	≥18	0.64 (0.37, 1.10)	0.11	0.78 (0.43, 1.41) ^3^	0.42
	≥65	0.79 (0.42, 1.46)	0.45	0.91 (0.47, 1.78) ^1^	0.79

^1^ Adjusted for sex, age, swab delay, and presence of respiratory disease. ^2^ Adjusted for sex, age, and presence of respiratory disease. ^3^ Adjusted for age, swab delay, season, presence of cardiovascular and respiratory diseases. aOR, adjusted odds ratio; CI: confidence interval; OR: unadjusted odds ratio.

## Data Availability

Raw data generated during the current study are not publicly available due to CIRI-IT internal policy and local ethical restrictions. Subject to certain criteria, conditions, and exceptions, and upon a reasonable request from qualified researchers, CIRI-IT may provide access to the data.

## Supplementary Materials

The following supporting information can be downloaded at: https://www.mdpi.com/article/10.3390/vaccines13101046/s1, Table S1: Full results of the real-time polymerase chain reaction (RT-PCR) (*N* = 188); Table S2: Association between count of signs and symptoms (total symptom score) and potential predictors: Sensitivity analysis by applying a multivariable linear model (*N* = 188); Table S3: Association between in-hospital mortality and potential predictors; and Table S4: Association between radiologically confirmed pneumonia and potential predictors.

## Abbreviations

The following abbreviations are used in this manuscript:

aOR	Adjusted odds ratio
ARI	Acute respiratory infection
CI	Confidence interval
Ct	Cycle threshold
ED	Emergency department
GP	General practitioner
ICU	Intensive care unit
IIV	Inactivated influenza vaccine
ILI	Influenza-like illness
IQR	Interquartile range
IV	Influenza vaccination
IVE	Influenza vaccine effectiveness
OR	Odds ratio
PR	Prevalence ratio
RSV	Respiratory syncytial virus
RT-PCR	Real-time polymerase chain reaction
SD	Standard deviation
