# Supplementary material for: Effect of Influenza Vaccination on the Disease Severity and Viral Load Among Adult Outpatients and Inpatients
Source: Vaccines (Basel). 2025 Oct 10;13(10):1046. doi: 10.3390/vaccines13101046 (PMC12567737; doi:10.3390/vaccines13101046)
Supplement: Supplementary file 1 [file vaccines-13-01046-s001.zip › vaccines-3903045-supplementary.pdf]

Table S1. Full results of the real-time polymerase chain reaction (RT-PCR) (*N* = 188).

**Pathogens Detected**	***n* (%)**
A(H1N1)pdm09	73 (38.8)
A(H3N2)	52 (27.7)
Influenza B	13 (6.9)
A(H1N1)pdm09 + Influenza B	1 (0.5)
A(H1N1)pdm09 + *Streptococcus pneumoniae*	7 (3.7)
A(H3N2) + *Hemophilus influenzae*	6 (3.2)
A(H1N1)pdm09 + *Hemophilus influenzae*	5 (2.7)
A(H3N2) + *Streptococcus pneumoniae*	3 (1.6)
Influenza B + *Streptococcus pneumoniae*	3 (1.6)
A(H1N1)pdm09 + *Streptococcus pneumoniae* + *Hemophilus influenzae*	3 (1.6)
A(H1N1)pdm09 + Coronavirus OC43	2 (1.1)
A(H3N2) + Metapneumovirus	2 (1.1)
A(H3N2) + Rhinovirus	2 (1.1)
A(H1N1)pdm09 + Rhinovirus	1 (0.5)
A(H1N1)pdm09 + Respiratory syncytial virus B	1 (0.5)
A(H1N1)pdm09 + Coronavirus 229E	1 (0.5)
A(H1N1)pdm09 + SARS-CoV-2	1 (0.5)
A(H1N1)pdm09 + Mycoplasma pneumoniae	1 (0.5)
A(H3N2) + Respiratory syncytial virus A	1 (0.5)
A(H3N2) + Adenovirus	1 (0.5)
A(H3N2) + Coronavirus OC43	1 (0.5)
A(H3N2) + SARS-CoV-2	1 (0.5)
A(H1N1)pdm09 + Metapneumovirus + *Hemophilus influenzae*	1 (0.5)
A(H1N1)pdm09 + *Mycoplasma pneumoniae* + *Hemophilus influenzae*	1 (0.5)
A(H3N2) + *Streptococcus pneumoniae* + *Hemophilus influenzae*	1 (0.5)
A(H3N2) + Respiratory syncytial virus A + *Streptococcus pneumoniae*	1 (0.5)
A(H3N2) + Rhinovirus + *Hemophilus influenzae*	1 (0.5)
A(H3N2) + Metapneumovirus + Coronavirus OC43	1 (0.5)
A(H3N2) + Bocavirus + *Streptococcus pneumoniae* + *Hemophilus influenzae*	1 (0.5)

Table S2. Association between count of signs and symptoms (total symptom score) and potential predictors: Sensitivity analysis by applying a multivariable linear model (N = 188).

	***b* (95% CI)**	***P***
Influenza vaccination	–0.92 (–0.12, –1.71)	0.025
Age (10-year increase)	–0.40 (–0.78, –0.03)	0.037
Season (2024/2025 vs 2023/2024)	–0.63 (–1.39, 0.13)	0.11
Cardiovascular disease	0.04 (–0.78, 0.86)	0.92
Smoking	0.84 (–0.20, 1.87)	0.11

CI: confidence interval.

Table S3. Association between in-hospital mortality and potential predictors.

Variable	≥ 18 years (*N* = 281)				≥ 65 years (*N* = 198)			
	Univariable Models		Multivariable model		Univariable Models		Multivariable model	
	OR (95% CI)	*P*	OR (95% CI)	*P*	OR (95% CI)	*P*	OR (95% CI)	*P*
Influenza vaccination	0.58 (0.20, 1.45)	0.26	0.37 (0.12, 0.97)	0.042	0.40 (0.13, 1.01)	0.053	0.36 (0.12, 0.94)	0.036
Sex (male vs female)	0.54 (0.22, 1.23)	0.14	0.58 (0.24, 1.37)	0.22	0.55 (0.22, 1.30)	0.18	0.59 (0.24, 1.43)	0.25
Age (10-year increase)	1.78 (1.24, 2.78)	< 0.001	1.85 (1.26, 2.93)	0.001	1.45 (0.82, 2.65)	0.20	1.54 (0.88, 2.81)	0.13
Swab delay (1-day increase)	1.21 (0.96, 1.53)	0.11	1.08 (0.84, 1.39)	0.57	1.12 (0.88, 1.42)	0.36	–	–
Season (2024/2025 vs 2023/2024)	0.74 (0.32, 1.71)	0.47	–	–	0.67 (0.28, 1.63)	0.37	–	–
≥ 1 co-morbidity	0.93 (0.36, 2.76)	0.88	–	–	0.51 (0.17, 1.77)	0.27	–	–
Cardiovascular disease	1.08 (0.47, 2.70)	0.85	–	–	0.56 (0.22, 1.49)	0.23	–	–
Respiratory disease	2.14 (0.88, 4.98)	0.093	2.23 (0.87, 5.51)	0.093	2.26 (0.90, 5.47)	0.080	2.22 (0.87, 5.46)	0.095
Diabetes	1.39 (0.47, 3.57)	0.53	–	–	1.14 (0.37, 2.98)	0.81	–	–
Renal disease	1.92 (0.57, 5.34)	0.27	–	–	1.43 (0.42, 4.04)	0.54	–	–
Cancer/immunosuppression	0.56 (0.06, 2.30)	0.47	–	–	0.74 (0.08, 3.26)	0.73	–	–
A(H3N2) vs A(H1N1)pdm09	0.98 (0.41, 2.26)	0.97	–	–	0.85 (0.34, 2.01)	0.71	–	–
B vs A(H1N1)pdm09	0.39 (0.003, 3.25)	0.46	–	–	0.61 (0.005, 5.82)	0.72	–	–

CI: confidence interval; OR: odds ratio.

Table S4. Association between radiologically confirmed pneumonia and potential predictors.

Variable	≥ 18 years (N = 281)				≥ 65 years (N = 198)			
	Univariable Models		Multivariable Model		Univariable Models		Multivariable Model	
	OR (95% CI)	*P*	OR (95% CI)	*P*	OR (95% CI)	*P*	OR (95% CI)	*P*
Influenza vaccination	0.64 (0.37, 1.10)	0.11	0.78 (0.43, 1.41)	0.42	0.79 (0.42, 1.46)	0.45	0.91 (0.47, 1.78)	0.79
Sex (male vs female)	1.14 (0.70, 1.86)	0.60	–	–	1.55 (0.84, 2.91)	0.16	1.59 (0.84, 3.07)	0.15
Age (10-year increase)	0.77 (0.66, 0.90)	0.001	0.83 (0.69, 1.01)	0.064	0.50 (0.33, 0.75)	< 0.001	0.53 (0.34, 0.82)	0.004
Swab delay (1-day increase)	0.80 (0.69, 0.93)	0.003	0.83 (0.71, 0.98)	0.025	0.86 (0.72, 1.02)	0.080	0.91 (0.76, 1.10)	0.33
Season (2024/2025 vs 2023/2024)	0.69 (0.42, 1.14)	0.15	0.70 (0.41, 120)	0.20	0.69 (0.37, 1.30)	0.25	–	–
≥ 1 co-morbidity	0.92 (0.51, 1.70)	0.79	–	–	0.85 (0.34, 2.26)	0.73	–	–
Cardiovascular disease	0.71 (0.43, 1.17)	0.18	1.01 (0.56, 1.85)	0.98	1.03 (0.50, 2.22)	0.94	–	–
Respiratory disease	2.11 (1.21, 3.71)	< 0.001	2.00 (1.11, 3.61)	0.021	2.37 (1.20, 4.65)	0.013	2.41 (1.19, 4.87)	0.014
Diabetes	0.66 (0.32, 1.28)	0.22	–	–	0.75 (0.33, 1.59)	0.47	–	–
Renal disease	0.80 (0.34, 1.75)	0.59	–	–	0.99 (0.40, 2.28)	0.98	–	–
Cancer/immunosuppression	1.57 (0.70, 3.45)	0.27	–	–	0.93 (0.27, 2.74)	0.90	–	–
A(H3N2) vs A(H1N1)pdm09	0.94 (0.56, 1.56)	0.80	–	–	1.16 (0.62, 2.16)	0.63	–	–
B vs A(H1N1)pdm09	0.96 (0.27, 3.02)	0.94	–	–	0.84 (0.08, 4.78)	0.86	–	–

CI: confidence interval; OR: odds ratio.
